# Upfront dilution of ferritin samples to reduce hook effect, improve turnaround time and reduce costs

**DOI:** 10.11613/BM.2018.010903

**Published:** 2017-11-24

**Authors:** Shu Juan Wu, Joshua A. Hayden

**Affiliations:** 1 Central Laboratory, Weill Cornell Medical College, New York Presbyterian Hospital, New York, USA; 2 Department of Pathology and Laboratory Medicine, Weill Cornell Medical College, New York, USA

**Keywords:** immunoassay, prozone effect, ferritin, turnaround time

## Abstract

**Introduction:**

Sandwich immunoassays offer advantages in the clinical laboratory but can yield erroneously low results due to hook (prozone) effect, especially with analytes whose concentrations span several orders of magnitude such as ferritin. This study investigated a new approach to reduce the likelihood of hook effect in ferritin immunoassays by performing upfront, five-fold dilutions of all samples for ferritin analysis. The impact of this change on turnaround time and costs were also investigated.

**Materials and methods:**

Ferritin concentrations were analysed in routine clinical practice with and without upfront dilutions on Siemens Centaur® XP (Siemens Healthineers, Erlang, Germany) immunoanalysers. In addition, one month of baseline data (1026 results) were collected prior to implementing upfront dilutions and one month of data (1033 results) were collected after implementation.

**Results:**

Without upfront dilutions, hook effect was observed in samples with ferritin concentrations as low as 86,028 µg/L. With upfront dilutions, samples with ferritin concentrations as high as 126,050 µg/L yielded values greater than the measurement interval and would have been diluted until an accurate value was obtained. The implementation of upfront dilution of ferritin samples led to a decrease in turnaround time from a median of 2 hours and 3 minutes to 1 hour and 18 minutes (P = 0.002).

**Conclusions:**

Implementation of upfront dilutions of all ferritin samples reduced the possibility of hook effect, improved turnaround time and saved the cost of performing additional dilutions.

## Introduction

While sandwich immunoassays offer a number of advantages in the clinical laboratory they also have a number of disadvantages, such as the potential for falsely low results due to hook (prozone) effect (*1*). Hook effect describes the paradoxical loss of signal that can occur in sandwich immunoassays in the presence of high concentrations of analyte. Because of this, erroneously low results can be reported by the clinical laboratory. The hook effect is most commonly encountered with analytes that span a wide range of concentrations, such as those secreted by tumours including serum free light chains and prolactin (*2*). Hook effect has also been reported in a number of ferritin immunoassays (*3*). Ferritin is an acute phase reactant and patients can have concentrations orders of magnitude above those commonly seen. High ferritin concentrations are especially common in macrophage activation syndrome and adult onset Stills disease (*4*). While rare, patients with these conditions can be encountered in tertiary care facilities such as ours. In these cases, erroneously reporting low ferritin concentrations can lead to inappropriate patient care.

Clinical laboratories have sought to detect hook effect proactively through a variety of means. Some have instituted a separate orderable for when there is clinical suspicion hook effect might occur (*5*). Others have employed neural learning networks to evaluate reaction curves and detect hook effect (*6*). With regards to lateral flow assays, recent efforts have been reported that investigate reaction kinetics to reduce hook effect (*7*). Finally, laboratories have performed automatic re-analysis of ferritin concentrations that fall within the hook range on dilution (*3*). This short communication details our medical centre’s experience in implementing upfront dilution of all samples to reduce hook effect in ferritin assays. These dilutions were performed automatically by the immunoassay analyser for all samples.

## Materials and methods

### Study design

A retrospective, descriptive study was performed to determine the impact of implementing upfront dilution of all ferritin samples. Prior to this change, all ferritin samples were analysed undiluted. Any ferritin result which fell above the upper limit of the measurement interval (1650 ug/L) had to be retrieved, diluted and re-analysed till an accurate value was obtained. Implementation of the upfront dilutions meant that all samples were diluted five-fold prior to analysis; no samples were analysed undiluted.

### Subjects

This study was performed at Weill Cornell Medical Center between April and May of 2017. All patients with ferritin measured in our laboratory as part of routine clinical care were included. No samples were collected for this study and no individually identifiable information was recorded. Thus, according to U.S. Department of Health and Human Services, this research is not research involving human subjects (*8*). Three sample sets were included in this study. The first set included two samples for which clinicians had expressed concern hook effect was present. These samples were initially tested without upfront dilutions and re-tested with upfront dilutions at clinician request. The second sample set included 1026 ferritin results reported in the month prior to implementing upfront dilutions and the third set included 1033 ferritin results reported after implementing upfront dilutions. These sample sets were compared with respect to turnaround time and number of samples that needed to be retrieved for dilution.

### Methods

Following hospital policies, samples were collected in serum separator tubes (Becton, Dickinson and Company, Franklin Lakes, USA) and sent to the clinical laboratory where samples were centrifuged for 6 minutes at 1839xg and analysed immediately. Ferritin testing was performed on a Siemens Centaur® XP (Siemens Healthineers, Erlangen, Germany). The coefficient of variation (CV) for this assay was under 6% and the reference intervals were 22 – 322 µg/L for men and 10 – 291 µg/L for women. Throughout the study period, all quality assurance protocols were followed in the clinical laboratory including three levels of control every twelve hours (BioRad, Hercules, USA). All instruments were operated by certified medical laboratory scientists.

For the upfront dilutions, the Centaur® XP was programmed to automatically perform a five-fold dilution using ADVIA Centaur Multi-Diluent 1 (Siemens Healthcare Diagnostics, Eschborn, Germany) prior to analysis. No samples were analysed undiluted on the instrument. This upfront dilution meant that the measuring interval went from 0.5 – 1650 µg/L to 2.5 – 8250 µg/L. This change was necessitated by the upfront five-fold dilution.

Turnaround time was calculated from the time samples were received in the laboratory to the time results were reported.

### Statistical analysis

All data were analysed in Microsoft Excel (Microsoft, Redmond, USA). The turnaround times for ferritin results before and after implementing upfront dilutions were compared to determine if they differed significantly. The turnaround times were checked for normality using the Shapiro-Wilk test of normality. Since the data were not normally distributed, comparisons were done using the independent two-group Mann–Whitney U test to compare the turnaround times before and after implementation of the upfront dilutions. All statistical calculations were performed using the program R (The R Foundation, Vienna, Austria).

## Results

### Reduction in hook effect

Two samples during the study period were identified that had very high ferritin concentrations (86,028 µg/L and 126,050 µg/L). Without upfront dilutions, both of these samples yielded erroneously low concentrations within the measurement interval and would have been erroneously reported out as 1495 µg/L and 1020 µg/L. When these same samples were tested with upfront dilutions, they returned concentrations above the upper limit of the measurement interval and prompted further dilutions. Thus, the upfront dilutions prevent hook effect in samples with ferritin concentrations up to 126,050 ug/L. Unfortunately, higher ferritin concentrations were not available to test the hook effect boundary.

### Turnaround time

**Figure 1:**
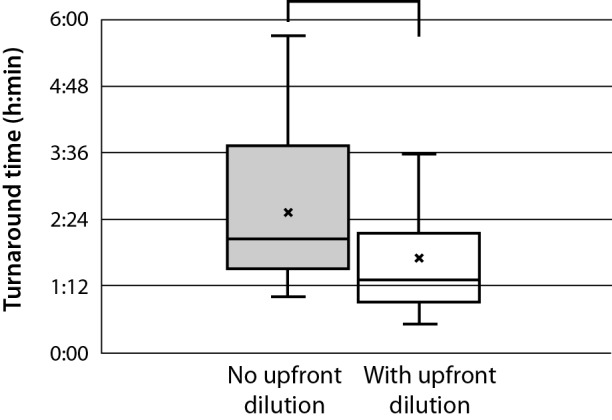
Turnaround time for ferritin concentrations greater than 1650 µg/L without (N = 37) and with (N = 34) upfront dilutions. A decrease in turnaround time was noted for these samples upon introduction of upfront dilutions (P = 0.002).

The overall turnaround time for all ferritin results before and after implementation of upfront dilutions was compared. Before implementation, the median turnaround time was 1 hour and 4 minutes (N = 1026). After implementation, the median turnaround time was 55 minutes (N = 1033). The Shapiro-Wilk test of normality showed that the data were not normally distributed and so they were compared with the independent two-group Mann–Whitney U test, which showed they were not significantly different (P > 0.05). Since the implementation of upfront dilutions was most likely to impact the turnaround time of samples with high ferritin concentrations, the turnaround times for samples with ferritin results greater than 1650 µg/L was compared before and after implementation of upfront dilutions as shown in Figure 1. Before implementation of the change, there were 37 samples with high ferritin concentrations and the median turnaround time was 2 hours and 3 minutes (interquartile range of 133 minutes). After the change, there were 34 samples with high ferritin concentrations and the median turnaround time was 1 hour and 18 minutes (interquartile range of 73 minutes). These data were not normally distributed using the Shapiro-Wilk test but the Mann-Whitney U test showed there was a statistically significant decrease in the turnaround time after implementation of the upfront dilutions (P = 0.002).

### Reduction in costs

Without upfront dilutions, 37 of the 1026 samples had results above the upper limit of the measurement interval and required dilution to report an accurate result. With upfront dilution, only 4 of the 1033 samples needed to be diluted to obtain an accurate result. Workflow estimates showed that the process of retrieving samples, making the dilutions and re-running the samples took an average of 20 minutes. Since there were 34 fewer dilutions with upfront dilutions, this saved 11 person hours (34 dilutions x 20 minutes).

## Discussion

Without upfront dilutions, it was found that samples with ferritin concentrations at 86,028 µg/L displayed hook effect; this is consistent with the manufacturer’s studies, which report no hook effect below 80,000 µg/L. However, this level of hook effect is smaller than what has been reported in the literature (*3*). It is unclear why there is such a discrepancy. With upfront dilutions, hook effect was not observed in a sample with ferritin concentrations as high as 126,050 µg/L. Unfortunately, we were unable to test the boundary of the hook effect with upfront dilutions. However, the chance of encountering ferritin concentrations above this are exceedingly rare. Other approaches to overcome hook effect have been proposed. Others reported using a separate orderable that tells the lab to perform dilutions (*5*). The disadvantage of this approach is that it requires proper selection of the orderable by the clinical teams. Our approach avoids this by having upfront dilutions programmed on the instrument. In addition, our approach is simple and does not require the expertise to implement more complex approaches, such as neural learning networks (*6*).

A limitation of this study is that this is a single center study peformed during a limited time period. In addition, a small percent of patients benefit from this approach. Finally, running each sample diluted instead of undiluted impacts the lower limit of the measurement interval; however, the difference between a lower limit of 0.5 and 2.5 ug/L is unlikely to have clinical significance. In conclusion, upfront dilutions are one approach to overcome hook effect in ferritin assays. This approach improves turnaround time for high ferritin concentrations and reduces costs.
